# Visual Information Pianists Use for Efficient Score Reading

**DOI:** 10.3389/fpsyg.2018.02192

**Published:** 2018-11-22

**Authors:** Eriko Aiba, Yutaka Sakaguchi

**Affiliations:** ^1^Department of Mechanical and Intelligent System Engineering, Graduate School of Informatics and Engineering, University of Electro-Communications, Tokyo, Japan; ^2^Center for Art and Performance Science, University of Electro-Communications, Tokyo, Japan

**Keywords:** sight-reading, pianist, readable range, geometrical features, appearance probability

## Abstract

When sight-reading music, pianists have to decode a large number of notes and immediately transform them into finger actions. How do they achieve such fast decoding? Pianists may use geometrical features contained in the musical score, such as the distance between notes, to improve their efficiency in reading them. The aim of this study is to investigate the visual information pianists rely on when reading music. We measured the accuracy of the musical score reading of 16 skilled pianists and investigated its relationship with the geometrical features. When a single note was presented, pianists easily read it when it was located within three ledger lines. When two notes with an octave interval were presented, interestingly, their readable range was extended compared to that of the single note. The pianists were also able to recognize the octave interval correctly even if they misread the height (or pitch) of the target notes. These results suggest that the pianists decoded two notes composing an octave interval as a single “two-tone geometric pattern.” Analyzing the characteristics of incorrect responses, we also found that pianists used the geometrical features of the spatial relationship between the note head and the ledger line, and that the cause of the misreading could be categorized into four types: [Type I] Confusion to a neighboring note having the same ledger line configuration; [Type II] Interference from a commonly used height note having the same note name; [Type III] Misunderstanding based on the appearance probability; [Type IV] Combination of the above three. These results all indicate that the pianists' abilities in score reading rely greatly on the correlation between the geometric features and playing action, which the pianists acquired through long-time training.

## Introduction

The musical score provides us various information necessary for musical performance, including not just the sequence of notes but note height and note value; performance expressions such as tempo, dynamics, and articulation; and performance techniques such as fingering and pedaling. In spite of such a huge amount of information, professional musicians can immediately process it and play the music.

Particularly, the sight-reading of piano music requires the processing of an enormous amount of information, as piano music includes many notes and chords written over the great staff. For example, Mozart's Sonata in A Major, K.331 (“Turkish March”) includes 6,182 notes in about 18-min performance and the first movement of Grieg's Piano Concert in A Minor, op.16, includes 6,600 notes in about 12-min performance (Hatano, 1987). In addition, pianist's brain must not only decode the musical notes, but also control their motor actions. The sight-reading process could be roughly described as follows: read the musical score; interpret the music; locate the keys to play while planning and controlling the finger motion. In addition, pianists must adjust the sound intensity and extension, sometimes using the sustain pedal. All this processing is performed simultaneously and continuously. To accomplish such complicated tasks in a limited time, it is important to handle various information efficiently. This research focuses on the mechanism of visual information processing realizing such efficient score reading.

Score reading is the very first process in playing the music, and its efficiency must have direct influence on the skills of the musical performance. Several studies investigated the process of score reading focusing on eye movements (Goolsby, 1994; Truitt et al., 1997; Waters et al., 1998). Goolsby (1994) reported that good sight-readers did not fixate on all the notes, whereas poor sight-readers tended to fixate on individual notes. Truitt et al. (1997) reported that better sight-readers had shorter fixation durations than poorer readers. Waters et al. (1998) showed that better sight-readers could read more notations within a single fixation. These results indicate that better sight-readers can process more information in a shorter time. In our previous experiment (Aiba and Matsui, 2016), the best sight-reader continued to read about 8.3 notes per second (in a short piece containing 581 notes), including other instruction marks. Our primary question is the visual processing mechanism for achieving such a rapid score reading.

**Figure 1:**
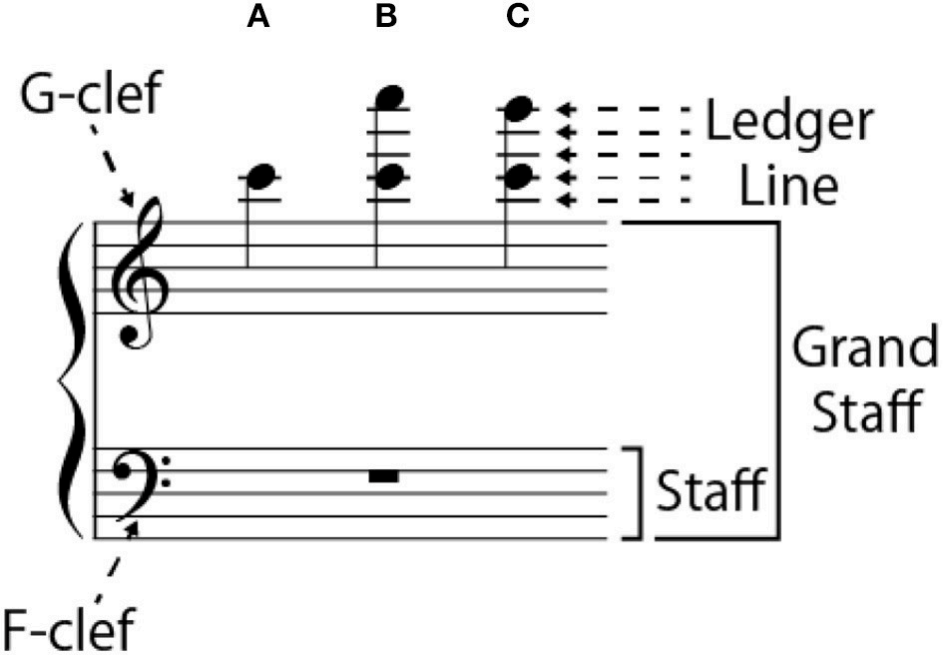
Terminology and example of target notes on G-clef: **(A)** single-note condition, **(B)** octave-interval condition, and **(C)** seventh-interval condition.

Some piano instruction books assert that using note patterns improves efficiency in score reading (Harris, 1998: Deutsch, 2013; Bradley and Tobin, 2016). For example, common patterns of multiple notes, such as octave, chord, scale, and arpeggio, could be used as clues to understand a larger number of notes at a glance. More specifically, sometimes the chord patterns help to read the score. For example, whereas it is somewhat difficult to quickly read the single C7 note shown in Figure 1A, it is relatively easy to read the C7 note in the octave interval (C6 + C7) shown in Figure 1B. On the contrary, when the notes indicating different heights are placed nearly at the same vertical position (as found in distorted handwritten scores) pianists are more likely to make mistakes. These empirical facts imply that pianists neither count the number of ledger lines outside of the musical staff, nor read individual notes in the chord. Instead, the pianists positively use geometrical features in the musical score such as the spatial relationship between notes and ledger lines, and the visual pattern of the chord. These findings suggest that the geometrical information (i.e., visual/spatial patterns) is more useful than the logical information (i.e., grammatical rules of music notation) for speeded score reading.

Considering the fact that the oldest existing musical score was written in the second century B. C., and that the modern musical notation was established around the seventeenth century (Grout et al., 2010), it can be said that musicians have developed legible musical notation through trial and error for over 2,000 years. These empirical facts mentioned above suggested that not only the legibility but also the geometrical patterns of the musical score are essential factors in score readability. However, to our knowledge, no experimental study has focused on this point.

In the present study, we measured the accuracy of the musical score reading of 16 skilled pianists and investigated its relationship with the geometrical features. All experimental stimuli (i.e., musical score) were correctly written in accordance with the actual modern notation method to eliminate the effect of distorted arrangement. We designed the experiment so that the pianists could respond in the usual piano playing manner.

The preliminary results of this study have been presented at the International Symposium on Performance Science 2017 (Aiba and Sakaguchi, 2017).

## Methods

### Participants

Sixteen pianists (2 male and 14 female) participated in the experiment. All had graduated from university musical programs with a degree in piano performance. Their ages ranged from 20 to 43 years old (mean ± *SD* = 26.7 ± 6.8) and their piano-training period ranged from 16 to 41 years (mean ± *SD* = 22.3 ± 6.6).

This experiment was approved by the University of Electro Communications Institutional Review Board for Human Subjects Research and was in accordance with the ethical standards stated in the Declaration of Helsinki. We obtained written informed consent from all the participants, and 5,200 Japanese Yen (about US$50) was paid to each for their participation.

### Apparatus

The participants played a hybrid piano (AvantGrand N2, YAMAHA) in a soundproof room. This hybrid piano generates sounds electronically but has the same mechanical key action as an acoustic grand piano. The sound signals were recorded from the hybrid piano to a notebook PC (X240, Lenovo) with desktop music software (SONAR X3 PRODUCER, TASCAM) through an audio interface (UA-1010, Roland) in a WAV format (16-bit, 48 kHz sampling rate) and MIDI. The musical score was presented on a LCD (FORIS FS2434, EIZO NANAO Co.) settled on the hybrid piano. The metronome sounds were presented from a speaker placed behind the display. The musical score and metronome sound presentation were operated by a computer program (Matlab R2013b, Mathworks) with Psychtoolbox 3 (Brainard and Vision, 1997; Pelli, 1997) through another notebook PC (Let's Note CF-S10, Panasonic). The stimulus (i.e., musical score) was generated by a music notation software (Finale 2012J, MakeMusic) in a JPEG image format.

### Range of target note

Three types of notes were served as target notes: a single note, an octave interval, and a seventh interval (Figure 1). The seventh interval was included to avoid the participants from being convinced that all target notes containing two notes had an octave interval.

**Figure 2:**
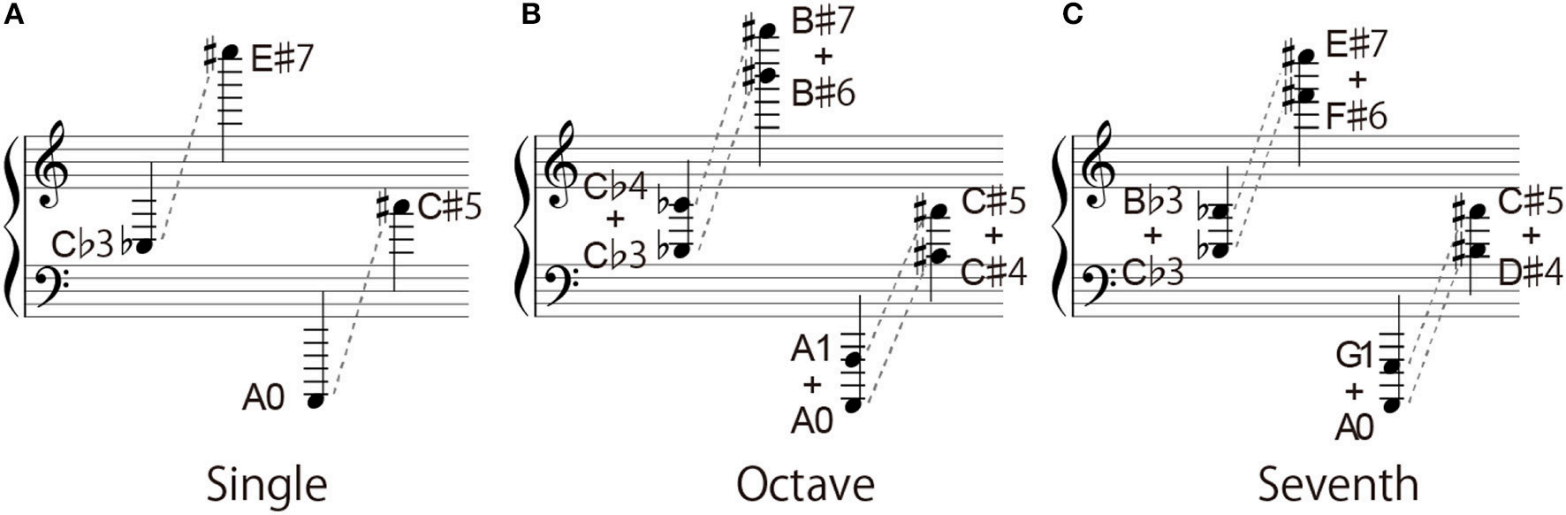
Range of target notes: **(A)** single-note condition, **(B)** octave-interval condition, and **(C)** seventh-interval condition.

For the single-note condition, the note height ranged from C♭3 to E♯7 on the G-clef, and from A0 to C♯5 on the F-clef (Figure 2A). E7 note has six ledger lines on the musical notation, and the A0 note (i.e., the left end key on the piano keyboard) has six ledger lines on the musical notation. For the octave interval condition, the height ranged from C♭3 + C♭4 to B♯6 + B♯7 on the G-clef, and from A0 + A1 to C♯4 + C♯5 on the F-clef (Figure 2B). B♯7 is the right end key on the piano keyboard. As for the seventh interval condition, its height ranged from C♭3 + B♭3 to F♯6 + E♯7 on the G-clef, and from A0 + G1 to D♯4 + C♯5 on the F-clef (Figure 2C). In the case of the octave and seventh interval conditions, the higher note and the lower note had either the same types of accidental marks (sharp and sharp/flat and flat) or without any accidental marks.

The total number of target notes was 251 in the G-clef and 238 in the F-clef (Table 1).

**Table 1:** Total number of target notes.

	**Single**	**Octave**	**Seventh**	**Total**
G-clef	93	84	74	251
F-clef	92	71	75	238

### Procedure

Prior to the experiment, participants' educational music experience was collected via a questionnaire.

**Figure 3:**
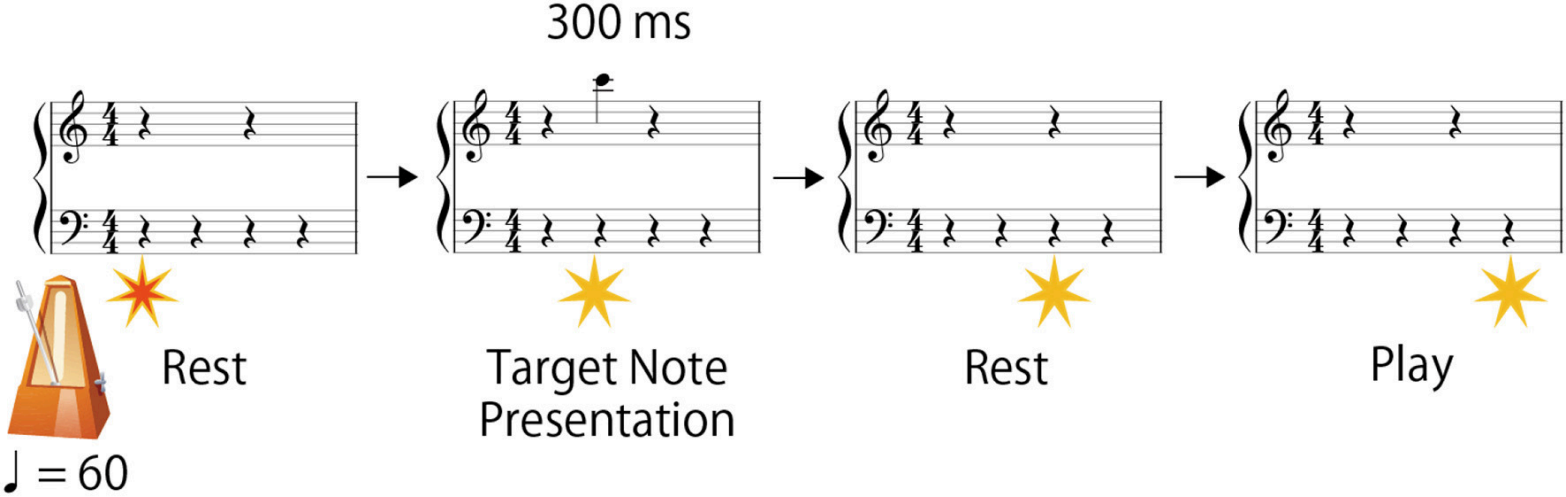
Flow of the experimental procedure.

Figure 3 illustrates the stimulus arrangement. The size of the grand staff was about the same size as the musical score for children (4.6 mm in height and 10.0 mm in width). In a trial, metronome sound was given at a fixed interval of 60 beats per minute (BPM 60) with the visual stimulus. The target notes were presented with the second metronome sound as quarter notes on a one-bar-length grand staff at the second beat position of a four-four time measure. The target notes were erased after 300 ms (i.e., presentation duration was 30% of the beat interval). Participants were asked to play the target notes at the fourth beat timing. The next trial started immediately after the present trial (i.e., no inter-trial pause).

Trials of the G-clef and the F-clef were performed in separate blocks, and the order of these blocks was counterbalanced among participants. Every target note was presented once in a randomized order to prevent participants from memorizing and learning them during the experiment. Accordingly, there were 251 and 238 trials in the G-clef and F-clef blocks, respectively. Each block was divided into three sessions. Participants could take a break if needed between the sessions. At the beginning of each session, a one-bar-length grand staff filled with quarter rests was presented (see Supplementary Material 1).

### Analysis

The participants' responses recorded in MIDI format were converted to Matlab data using the Matlab MIDI toolkit developed by Ken Schutte (Schutte, 2012).

The notes played during the period from 1,300 ms (i.e., the time of target note erase) to 4,000 ms (the end of the bar; the beginning of the next trial) were treated as the response for the presented trial. When the participants played multiple notes in the single-note condition, the first played note was used for further analysis. In the octave- and the seventh-interval conditions, in the same way, the first and the second played notes were used for further analysis. The order of the two key presses was ignored.

Because every target note was presented only once for every participant, we evaluated the correct response rate based on the pooled data. We set 80% (i.e., 13 out of 16 participants made correct responses) as a threshold for defining a readable range. This threshold was determined because the overall correct response rate was 78%.

## Results

### Overall performance

As mentioned above, overall correct response rate was 78%. The response time (i.e., difference between the fourth beat and the key-press time) was only 1.1 ms on average (±*SD* = 113.1 ms). There was no significant difference in response time between the correct responses (mean ± *SD* = 1.2 ± 100.0 ms) and the incorrect responses (mean ± *SD* = 0.7 ± 197.8 ms). This means that participants performed the experimental task faithfully as we instructed.

A logistic regression analysis was conducted on participants' response (correct vs. incorrect) with the accidental marks, the interval, and the clef conditions. The overall regression model was significant [X2 (11, *N* = 7584) = 368.66, *p* < 0.001]. The model detected a significant effect of the accidental mark and the interval conditions (Table 2). Among the intervals, the order of ease in correct responses was found to be in the octave > the single > the seventh. For the with/without an accidental mark, the correct response rate of the target note without an accidental mark was significantly higher than that of the target note with an accidental mark.

**Table 2:** Results of regression analysis.

**Condition**	**Correct response Rate (%)**	**Odds ratio (95% CI)**	***p*-value**
**ACCIDENTAL MARKS**			
Without	78.6	Reference	–
With	73.2	0.71 (0.63–0.81)	<0.001
**INTERVAL**			
Single	76.5	Reference
Octave	85.8	1.97 (1.68–2.33)	<0.001
Seventh	62.9	0.54 (0.48–0.62)	<0.001
**CLEF**			
G-clef	76.3	Reference
F-clef	73.7	0.91 (0.81–1.04)	n.s.

At a glance, the accidental mark seems to increase the processing load. However, when the accidental mark indicates to play a black key, the correct response rate was not significantly degraded compared to the target notes without accidental marks. However, when an accidental mark indicates to play a white key (i.e., B♯, E♯, C♭, and F♭), the correct response rate was lower than the other conditions. Further analysis on the accidental mark will be conducted in section Effects of Accidental Marks.

### Readable range

**Figure 4:**
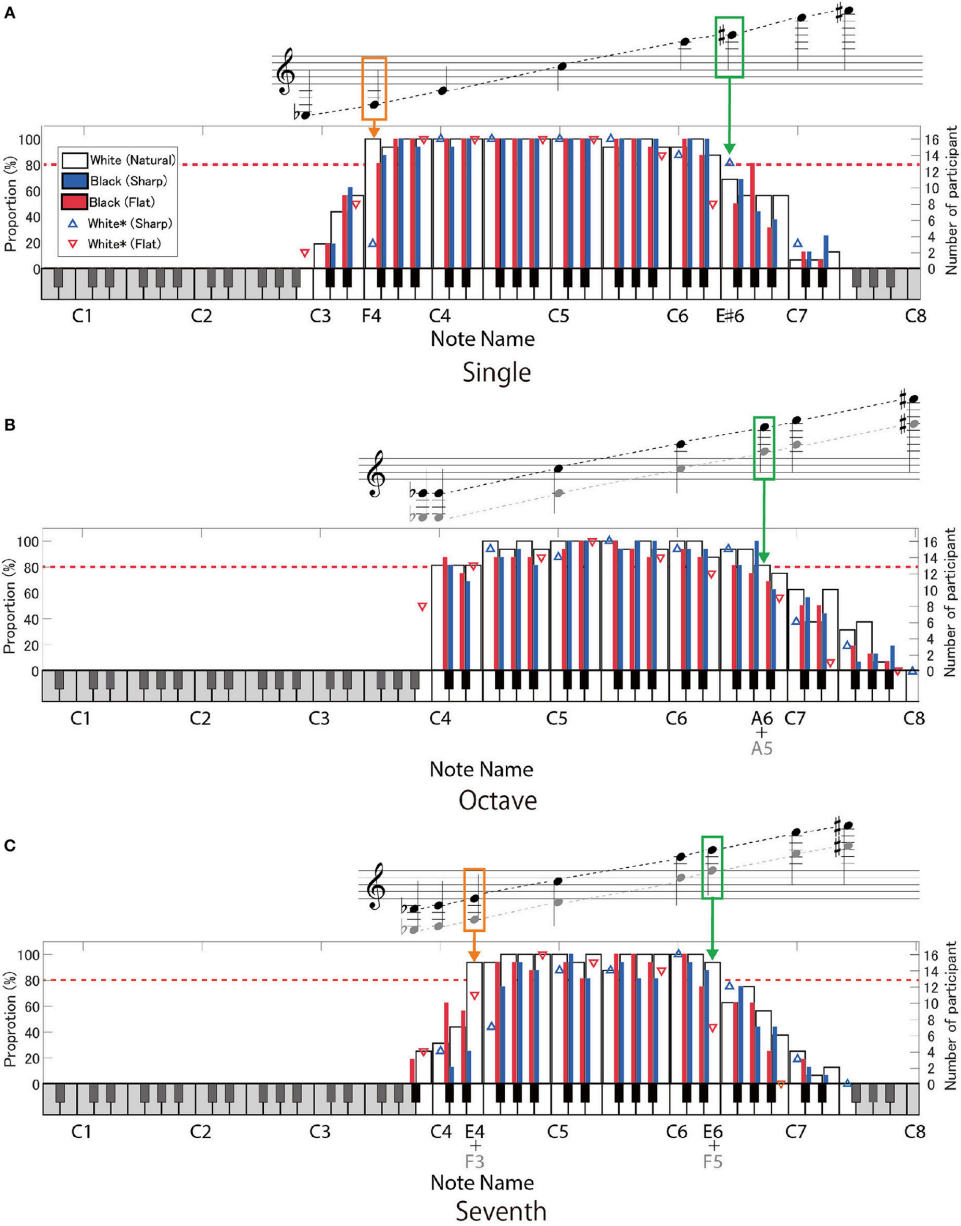
The proportion of the participants who gave correct answers to the target notes on the G-clef: **(A)** single-note, **(B)** octave-interval, and **(C)** seventh-interval conditions. The region of the target notes is indicated by the high-contrast area on the keyboard. In the octave **(B)** and the seventh intervals **(C)**, the note name (given in the lower part of the keyboard) indicates the “higher note” contained in the intervals (black-colored in the score). The white bars indicate the target notes without any accidental marks. The blue- and red-colored bars show the target notes with sharp (♯) or flat (♭), respectively, in the case that the accidental marks mean playing black keys. The blue- and red-colored triangles show the target notes with sharp and flat, respectively, in the case that the accidental marks indicate playing white keys (C♭, B♯, E♯, and F♭). The red dotted line indicates the 80% correct rate. The orange- and green-colored outlines indicate notes within the lower- and upper- limits of the readable notes (that is, where the correct response rate was over 80%), respectively.

**Figure 5:**
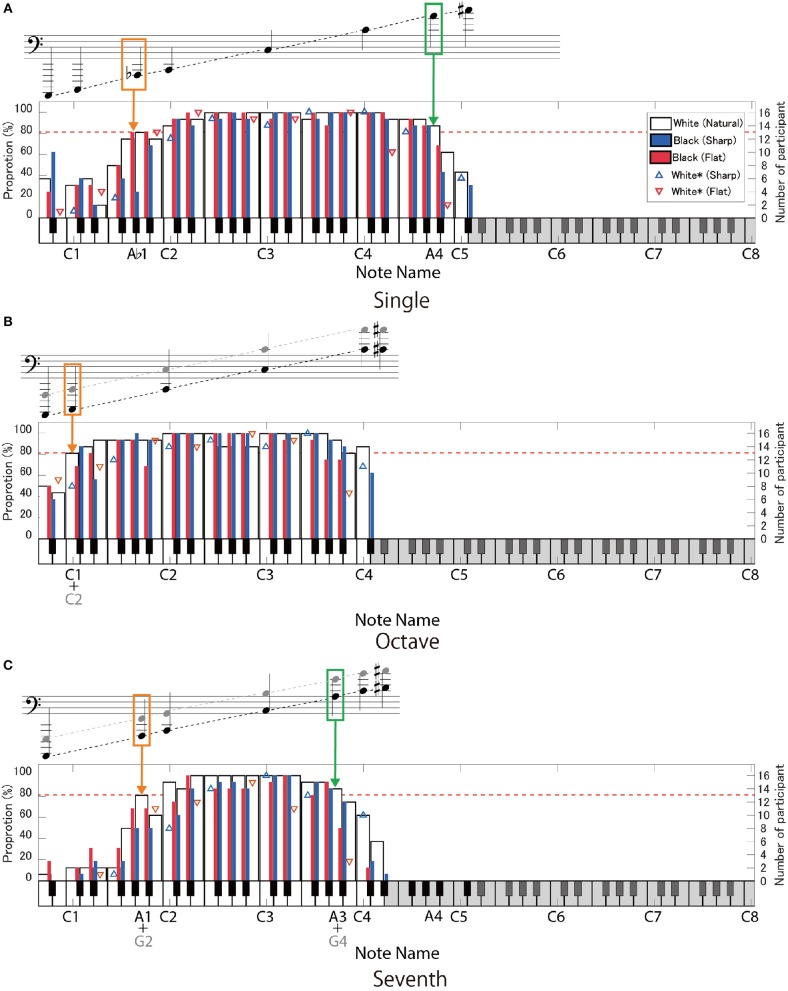
The proportion of the participants who gave correct answers to the target notes on the F-clef: **(A)** single-note, **(B)** octave-interval, and **(C)** seventh-interval conditions. The symbols mean the same as in Figure 4, except that the note name represents the “lower note” contained in the intervals **(B,C)**.

**Figure 6:**
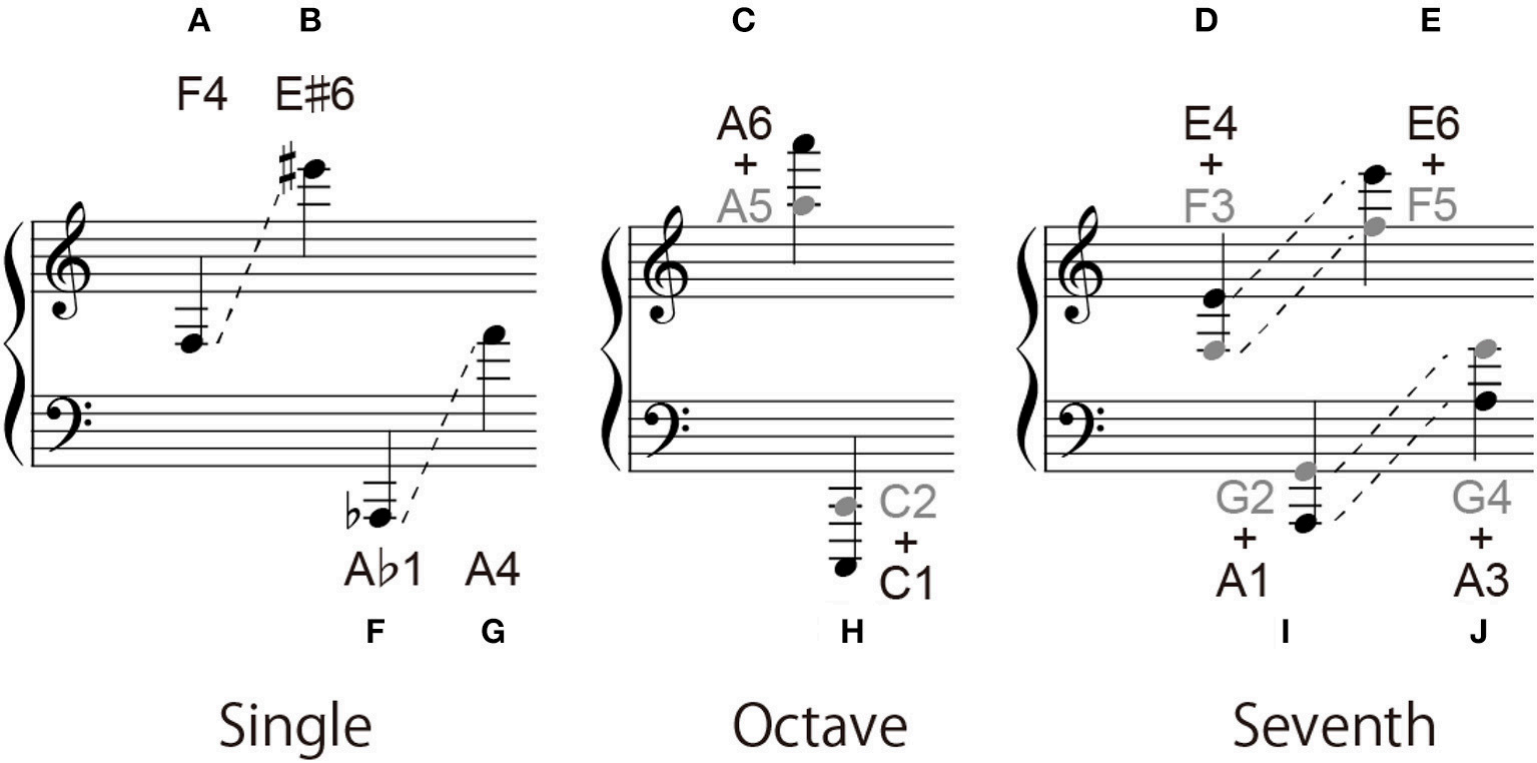
Readable range for each type of target note.

Figures 4, 5 show the correct response rates in three interval conditions. The red dotted line indicates the 80% threshold. In the G-clef for the octave- (Figure 4B) and the seventh-interval (Figure 4C) conditions, the note name corresponds to the higher note contained in the intervals (that is, C6 means C5 + C6 in the octave condition: the black-colored note name under the keyboard and the black-colored note in the musical score). In contrast, in the F-clef for the octave- (Figure 5B) and the seventh-interval (Figure 5C) interval conditions, the note name corresponds to the lower note contained in the intervals (that is, A1 means A1 + A2). In addition, the upper and lower limits of the readable range are showed in Figure 6.

#### Single note

As for the single-note condition on the G-clef, more than 80% of the participants made correct responses in the range of F3 to E♯6 (except for F♭3) (Figure 4A). In case of the F-clef, the target notes ranged from A♭1 to A4 and were correctly played by more than 80% of the time by participants (except for A♯1, B1, and B♯1) (Figure 5A). Interestingly, note that these boundary notes (i.e., F3 and E♯6 on the G-clef, and A♭1 and A4 on the F-clef) are all attached to the third ledger line!

Accidental marks little affected the response except for the cases playing the white keys (i.e., B♯, E♯, C♭ and F♭).

#### Octave intervals

As for the G-clef, the correct responses to the octave intervals were over 80% in the range from C3 + C4 to A5 + A6 (Figure 4B). In the case of the F-clef, the correct response rates reached 80% in the range from C1 + C2 to C3 + C4 (Figure 5B). Here, we should point out that the readable region in the octave-interval condition was wider than in the single-note condition: The boundary notes (i.e., C3 and A6 on the G-clef, and C1 and C4 on the F-clef) were attached to the fourth or fifth ledger lines, which are farther from the grand staff than the boundary of the single-note condition (i.e., the third ledger lines).

Note that the correct response rates were always over 80% in the middle part of the grand staff (i.e., the lower range on the G-clef and the higher range on the F-clef). Therefore, in the present experiment, we could not know the limit of the correct score reading in these conditions.

In addition, correct response rate was lowered with the accidental mark, in contrast to the single-note condition.

#### Seventh intervals

As for both the G-clef and the F-clef, the note between F3 + E4 and F5 + E6, and A1 + G2 and A3 + G4 were correctly answered by more than 80% of the pianists, respectively (except for B1 with or without accidental marks on the F-clef) (Figures 4C, 5C).

In addition, correct response rate was lowered with the accidental mark.

#### Summary and discussion

For the single-note condition, the participants were able to read up to the note on or above the third ledger line on both the G-clef and the F-clef. This readable range seems rather narrow considering that notes located farther than three ledger lines often appear in actual musical scores. This point will be discussed in section General Discussion.

As pointed out in section Octave Intervals, the participants were able to read the octave intervals even when they included more ledger lines than that of the readable single notes, in both the G-clef and F-clef conditions. This suggests that the participants decoded the two notes with an octave interval as a “two-tone geometric pattern.” Presumably, the note-to-note distance and spatial relationship to the ledger line may be regarded as essential features. Specifically, they interpreted that when one note of an octave interval is on the musical staff or on the ledger line, then the other note must be located between the musical staff or above/below the ledger line. Such an asymmetric pattern is a characteristic feature of an octave interval.

On the other hand, a note closer to the staff contained in an octave interval could not be readily read despite the same note being readable under the single-note condition. For example, the highest readable note on the G-clef was E♯6 (on the third ledger line) in the single-note condition but was A5 (on the first ledger line) + A6 in the octave condition. If the participants could make full use of positional information of notes closer to the staff, readable notes closer to the staff in the octave interval would agree with the highest readable single note. Therefore, this result suggests that some other factor prevented the participants from using completely the closer note information. This point will be discussed again in section Types of Incorrect Responses.

In the case of the seventh-interval condition, the readable range was consistent with the single-note condition: no extension was observed. We will further discuss this point in section Types of Incorrect Responses.

### Effects of accidental marks

**Figure 7:**
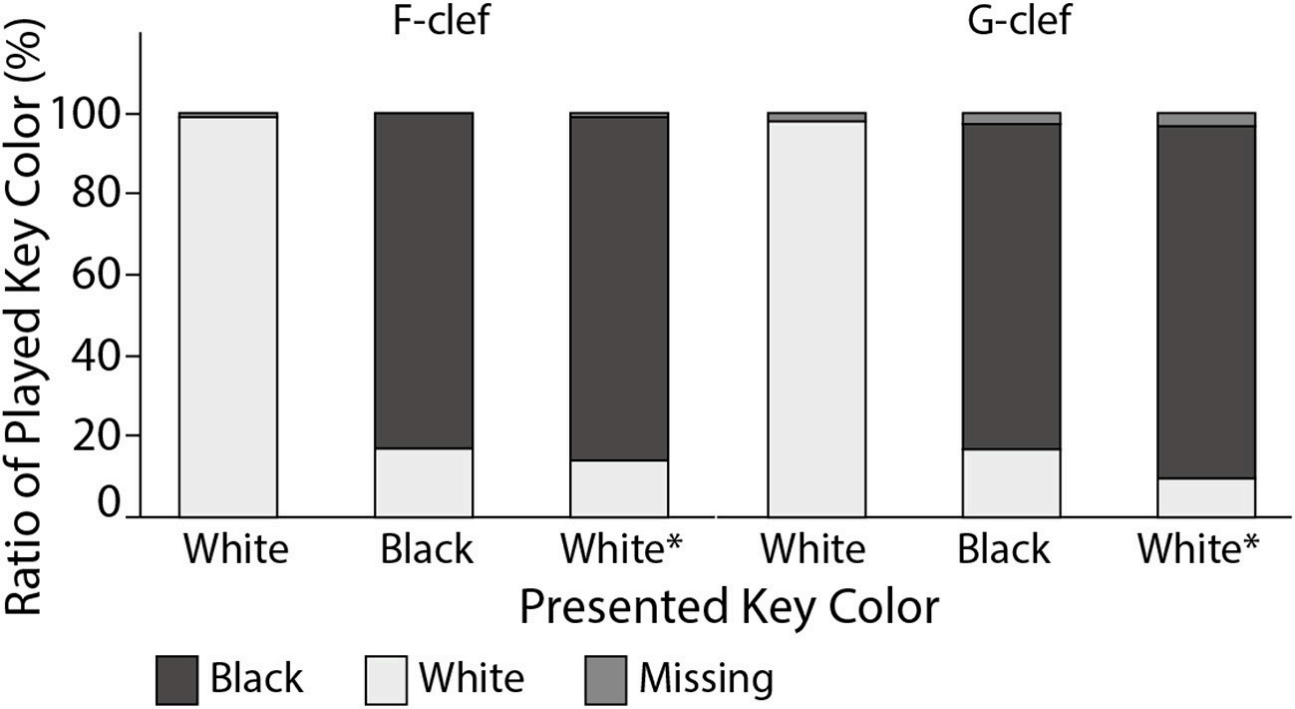
Ratios of played key color are indicated for each key color of the presented note, when the participants made incorrect responses under the single-note condition. The horizontal indexes “White”, “Black”, and “White*” represent the presented notes indicating white keys, black keys, and white keys with an accidental mark, respectively.

Figure 7 shows the ratio of the key colors (i.e., white vs. black) when the participants played an incorrect key under the single-note condition. The bar color indicates the played key color and missed (not played) key. The horizontal indexes “White,” “Black,” and “White^*^” represent the presented notes indicating white keys, black keys, and white keys with an accidental mark, respectively.

When no accidental mark was presented (White), the participants mostly played white keys, even when they played wrong keys. When the accidental mark indicated the need to play a black key (Black), the participants correctly played a black key 82.5% of the time. When the target note with an accidental mark indicated to play a white key (White^*^), the participants played a white key only in 13.0% of the trial. This result strongly suggests that accidental marks urge the pianists to press black keys.

These results mean that the presence and absence of accidental marks was an important clue to motor planning of piano performance.

### Types of incorrect responses

**Figure 8:**
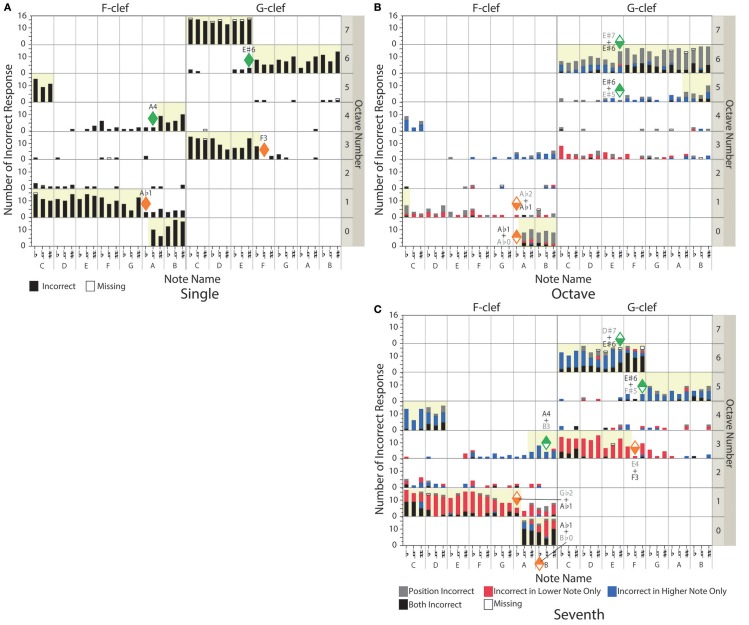
The number of incorrect responses is indicated for each target note: **(A)** single-note, **(B)** octave-interval, and **(C)** seventh-interval conditions. Classification of incorrect responses are indicated by the color of the bars. The horizontal indexes represent the presented note name, and the vertical indexes represent an octave number of the presented target note. The left and right panels indicate the target notes on the F-clef and the G-clef, respectively. In the case of the octave- and seventh-interval conditions, each note name and octave number corresponds to the lower note contained in the intervals. The orange- and green-colored diamonds represent notes within the lower- and upper- limits of the readable notes in the single note condition, respectively. The yellow background indicates the unreadable range.

Figure 8 shows the number of incorrect responses classified by the type of incorrect response. In the single-note condition (Figure 8A), incorrect responses were classified into “Position Incorrect” (i.e., the participants played incorrect keys) and “Missing” (i.e., the participants did not play any keys). In the octave-interval and the seventh-interval conditions, incorrect responses were classified into “Position Incorrect” (i.e., the participants played the correct interval at an incorrect height), “Incorrect in Lower Note Only” (i.e., the participants played incorrectly a lower note), “Incorrect in Higher Note Only” (i.e., the participants played incorrectly a higher note), “Both Incorrect” (i.e., the participants played the incorrect interval at an incorrect height), and “Missing.”

For the single note condition, the ratios of “Position Incorrect” and “Missing” responses were 23.6% and 0.3%, respectively.

For the octave-interval condition (Figure 8B), “Position Incorrect” was the major incorrect response. It means that the participants caught the correct interval even when they misread the notes' height. In the case that they had difficulty estimating the exact height (especially with notes accompanied by many ledger lines), the participants could immediately catch the exact interval, presumably based on the geometrical features of the target notes (such as the note-to-note distance and spatial relationship to the ledger lines).

From another point of view, the farther the target note went above the staff, the more “Incorrect in Higher Note Only” was observed (in other words, the note closer to the staff was correctly played). In the same manner, the farther the target note went below the staff, the more “Incorrect in Lower Note Only” was observed. These results suggest that the participants read the interval using the note closer to the musical staff. In addition, above F6♭ (+ F7♭), it seemed that the number of “Both Incorrect” increased instead of “Incorrect in Higher Note Only”. F6 is the note just above the threshold in the single-note condition [E♯6 (+ E♯7); see the green-colored diamond in Figure 8B]. This result also supports the inference that the participants read the interval using the note closer to the musical staff. However, even in this range, the major incorrect response was still “Position Incorrect.”

These results imply that there is some visuo-motor coupling between the geometric pattern of the octave interval and the width to splay the fingers. Further discussion of the incorrect responses on the intervals will be provided in section Differences Between the Presented Intervals and the Played Intervals.

For the seventh-interval condition (Figure 8C), incorrect responses belonged to mainly “Incorrect in Higher Note Only” and “Incorrect in Lower Note Only”. This means that the participants misread the interval, different from the octave condition. This point will be further analyzed in the next section.

However, as with the octave-interval condition, “Incorrect in Higher Note Only” increased as the target note went farther above the staff, and “Incorrect in Lower Note Only” increased as the target note went farther below the staff. As with the octave interval condition, the number of “Both Incorrect” drastically increased when the target notes reached the upper/lower limit of the single-note condition [i.e., E♯6 (+ D♯7) and A♭1 (+ G♭2); see the green-and orange-colored diamonds in Figure 8C].

### Differences between the presented intervals and the played intervals

**Figure 9:**
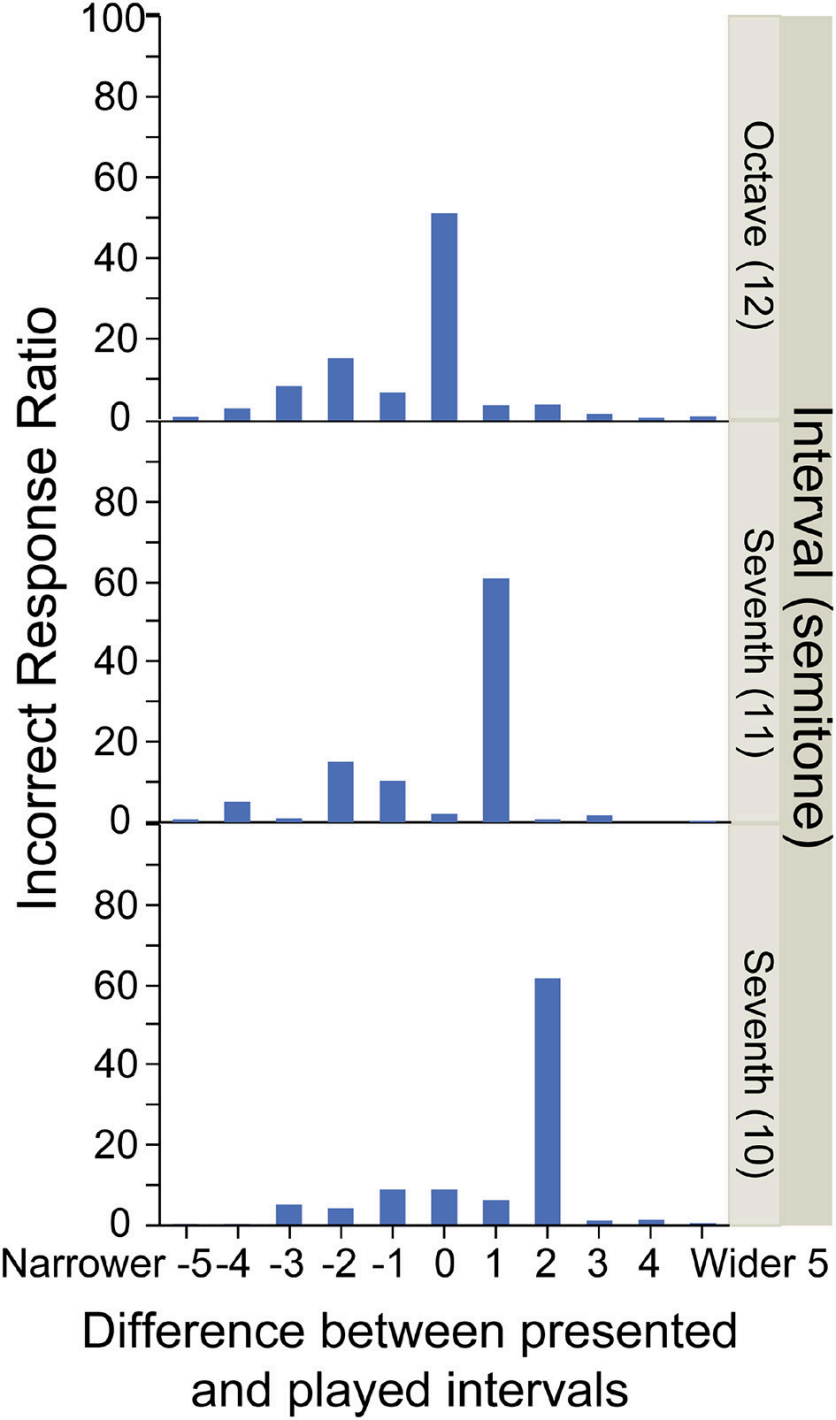
The ratio of errors in interval in the octave- and seventh-interval conditions. The amount of error was represented based on the unit of semitone.

Figure 9 summarizes the “interval error”—that is, the difference between the presented interval and the played interval for all incorrect responses (including the case in which only height was incorrect whilst the interval was correct). This figure shows the ratio of interval errors observed in the experiment, where the amount of error is represented by the unit of a semitone.

For the octave-interval condition, 51.7% of the incorrect responses had the correct interval. This data quantitatively supports the notion that the participants could often play the correct interval even when the height was incorrect. The second-most frequent mistake was the two-semitone narrower interval—that is, the seventh interval.

On the other hand, the seventh intervals were often played incorrectly as an octave interval. With regard to the seventh intervals with 11 semitones, 60.8% of the incorrect responses had a one-semitone-wider interval—that is, the octave interval. For the seventh intervals with 10 semitones, about 61.3% of incorrect responses had a two-semitone-wider interval—that is, the octave interval.

Although a seventh interval and an octave interval differ by only one note in distance, they are remarkably different in the ledger line configuration: two notes in a seventh interval have the same spatial relationship to the ledger lines (see Figures 6D,E,I,J), whilst two notes in an octave interval have a different relationship (see Figures 6C,H). Therefore, a seventh-interval should not be confused to an octave interval if the participants utilized this difference in the geometric feature. The result that the participants often played an octave interval for a seventh-interval note suggests the existence of another significant factor for misreading. A possible factor is the difference (or bias) in the appearance probability: Octave intervals frequently appear in the real piano score, but seventh-intervals do not.

### Differences between the presented notes and the played notes

In the single-note condition, the amount of positional error was concentrated in one or two notes. Below, we further analyzed the case that more than 20% of participants (i.e., 4 out of 16 participants) made the same positional error. The octave- and seventh-interval conditions were excluded from this analysis because various factors differed between two notes contained in both intervals.

Table 3 shows the list of the note names that the participants played incorrectly over 20% of the responses. The gray-colored row shows the note names with an accidental mark that instruct them to play a white key.

**Table 3:** List of unreadable note names in the single-note condition.

**G-clef**				**F-clef**			
**Presented note**	**♭**	**♮**	**♯**	**Presented note**	**♭**	**♮**	**♯**
C7	B♭6 (4)^*^	A6 (6)	A♯6 (6)	C5	B♭4 (7)	B4 (5)	A♯4 (9)
C3	D♭3 (6) E♭3 (4)	B2 (4) D3 (4)	D♯3 (7)	C1	B♭0 (5) D♭1 (4) E♭1 (4)	B0 (4) D1 (4)	A♯0 (4)
D7	B♭6 (4)	B6 (8)		D1	B♭0 (6)	F1 (4)	C♯1 (5) F♯1 (5)
D3	E♭3 (6) F♭3 (5)	F3 (4)				
E3	G♭3 (5)		F♯3 (5) G♯3 (4)	E1	F♯1 (4)	D1 (6)
E7	D♭7 (5)	C7 (4)				
F3	A♭3 (4)			F4	A♭4 (4)	
				F1	A♭1 (6)	A1 (4)	D♯1 (6)
G6	E♭6 (4)	E6 (4)		G1	A♭1 (4)		F♯1 (7)
A6			G♯6 (5)	A4			F♯4 (4) G♯4 (5)
				A0		C1 (4)
B6			A♯6 (4) C♯7 (4)	B0	D♭1 (7)	A0 (6) C1 (5)	A♯0 (5) C♯1 (6)
				B4		G4 (4)	G♯4 (4) A♯4 (4)

*The first column indicates the presented note name. The second to fourth columns represent a type of an accidental mark accompanied with the presented note. For example, the note name B♭6 in the first row of the second column indicates that four participants played B♭6 incorrectly when C♭7 was presented*.

*The grey-colored row shows the note names with an accidental mark that instruct them to play a white key*.

* *Numbers in parentheses mean the frequency*.

**Figure 10:**
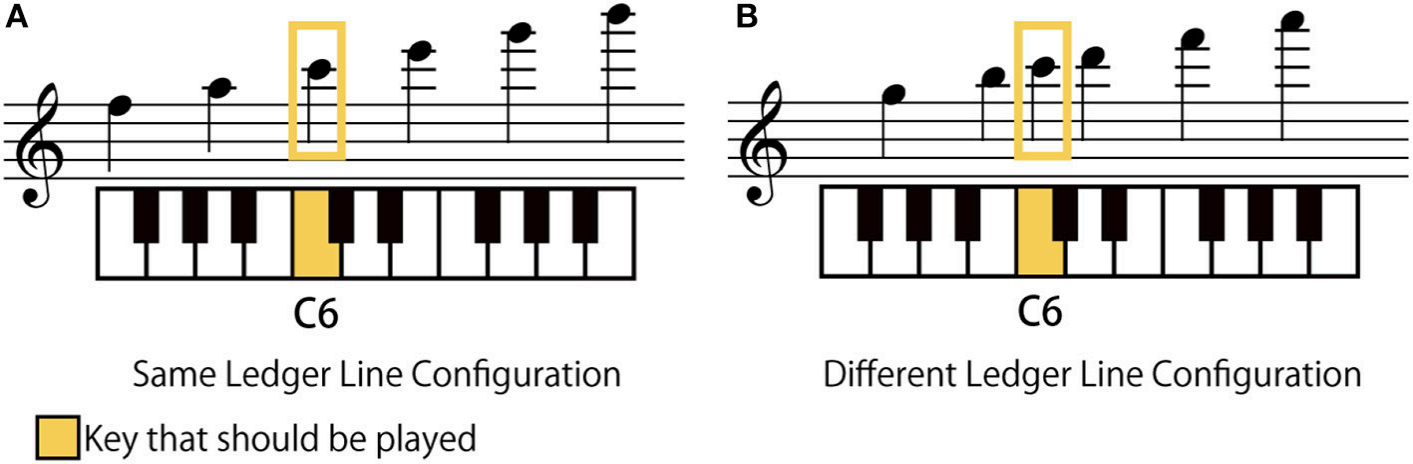
Example of the method of categorization of the C6 note. The notes in the left score **(A)** is on the ledger line that is the same as C6. The notes in the right score **(B)** above the ledger line that is different from C6.

**Figure 11:**
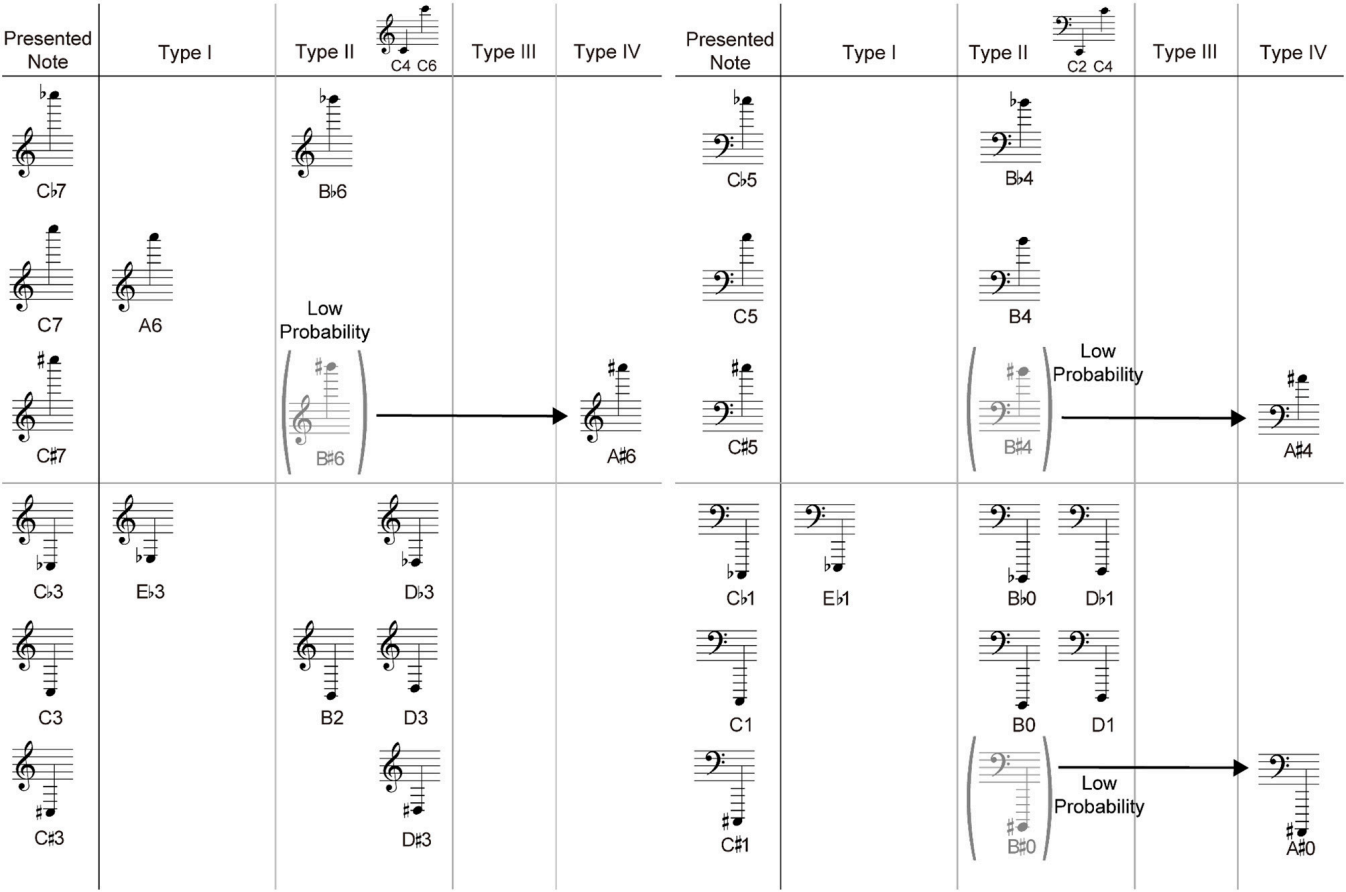
Example of positional errors to the C note.

We analyzed these positional errors in detail and found that their cause can be categorized into four types:

[Type I] Confusion to a note having the same ledger line configuration: The target note was played as a neighboring note having the same ledger line configuration (Figure 10). As we noted, the participants presumably judged the notes based on the ledger line configuration. Therefore, even though the participants misread the height of the target note, the ledger line configuration could be correctly read. For example, as shown in Type I of Figure 11, C notes were often misread as A or E notes.

[Type II] Interference from geometrical features in commonly used height range: The geometrical features affected the participants' responses. For example, as shown in Type II of Figure 11, C notes were often misread as B or D notes. In actual piano performance, C2, C4, and C6 are frequently played, and all these notes are located on the ledger line (i.e., the same ledger line configuration). Therefore, note C must be tightly associated with the note on the ledger line in the pianists' brains. Accordingly, when they encounter C1 and C7, which are below/above the ledger line, they would be less likely to recognize them as C, and as a result, they would incorrectly play adjacent key D or B.

**Figure 12:**
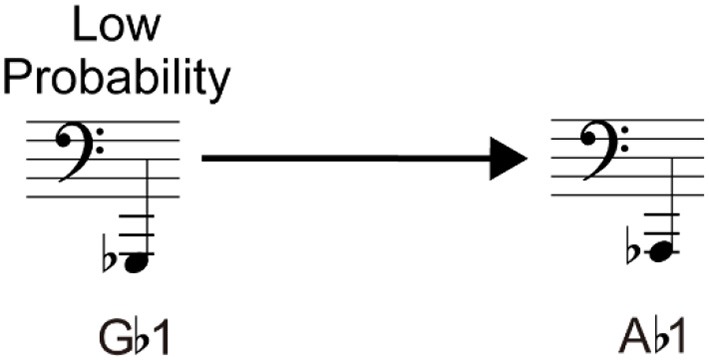
Example of a Type III error.

[Type III] Misunderstanding based on the appearance probability: The participants modified their responses according to the appearance probability of each note. For example, as shown in Figure 12, G♭1 on F-clef was often misread as A♭1. When A♭ and G♭ are compared, it seems that A♭ should appear more frequently based on the key signature. While A♭ appears in a key signature with three accidental marks, such as E♭ Major, G♭ appears in a key signature with five accidental marks, such as D♭ Major. We should note that the notes of exactly the same height either without an accidental mark or with sharp (i.e., G1 and G♯1) were correctly read in almost all the cases.

[Type IV] Combination of the above three: For example, as shown in Type IV of Figure 11, C♯ was often misread as A♯. This mistake may be classified to played was a Type I error. Actually, in the case of the C7 without an accidental mark, six participants misread it as A6 with the same ledger line configuration. However, this cannot be the only reason since C♭, C, and C♯ have identical geometrical features, except for the existence of an accidental mark. As mentioned above in the example of Type II, C♭/C are misread as B♭/B or D♭/D. It was therefore assumed that although the participants initially judged C♯ as B♯ based on the ledger line configuration, they changed their initial judgements based on the appearance probability, and misread C♯ as A♯.

Therefore, geometrical features and the appearance probability (or past experience) are the most influential factors in the score reading.

The results of classification on other notes will be provided as Supplementary Material 3.

## General discussion

In the present study, we showed that pianists could easily read a single note when it was located within three ledger lines. This readable range seems rather narrow considering that notes located farther than three ledger lines often appear in actual musical scores. For example, the scores of Chopin's Etudes, op. 10, includes many notes locating over the three ledger lines. A few possible reasons can be considered for this discrepancy. One is the difference between the experimental stimulus and real musical score. In the present experiment, only a target note was presented in an isolated manner whilst in the actual scores multiple notes are arranged in a sequence, as in scales and arpeggios. Pianists use such sequential (or temporal) patterns as clues for score reading. Another possible reason is the frequency of the ledger lines. In many scores, “octave signs” are used for reducing the number of ledger lines (and for improving the readability) if the note is far above or below the staff, and thus, the frequency that the pianists encounter the notes with many ledger lines is rather low. This low probability (or poor experience) might diminish the familiarity of the notes with many ledger lines and the development of their visual-motor mapping. In this sense, we may say that the establishment of the modern musical notation as a result of pursuing high legibility affected the pianists' ability of score-reading.

These results also support the view that learning to recognize note patterns, a common instruction tip, can lead to efficient score reading (Harris, 1998; Deutsch, 2013; Bradley and Tobin, 2016). Likewise, it supports the results of the previous studies that have showed that the speed of performing a pattern-matching exercise correlates with sight-reading skills (Waters et al., 1998; Kopiez and In Lee, 2008).

We also showed that the readable range of the octave interval was extended compared to that of the single note. In addition, the participants could recognize the correct interval, even if they misread their height. These results suggest that the pianists read two notes composing an octave interval as a single “two-tone geometric pattern.” They can estimate the interval from its geometric pattern, even if it is difficult for them to know the exact height.

The participants' incorrect responses for accidental marks support that they used appearance probability as an important clue. They played a black key over 80% of the time in both the Black and White^*^ conditions, although they played white keys in the White condition. There were only four White^*^ cases (i.e., C♭, B♯, F♭, and E♯) whilst the remaining ten cases were Black, that is, the ratio of the former case is about 28.6%. This ratio seems enough high to build visuo-motor association for White^*^. In the actual music, however, the ratio of White^*^ is much lower than this value because of the distribution of key signatures of the musical works. When considering the key signature, C♭, B♯, F♭, and E♯ appear in the key signature with six (G♭ Major), seven (C♯ Major), seven (C♭ Major), and six (F♯ Major) accidental marks, respectively. Although B♯ and E♯ often appear music in C♯ Minor and F♯ Minor as an accidental mark, the number of musical works written in these key signatures is relatively low. Actually, we counted the number of musical works for each key in the database of musical scores (International Music Score Library Project or IMSLP, online at http://imslp.org): The number of musical works in C♯ Minor and F♯ Minor was are only 201 and 309 out of 37,767, respectively (see Supplementary Material 2).

As for the intervals, it is assumed that the participants' incorrect responses were affected by appearance probability (i.e., past experience). The seventh intervals were often played incorrectly as an octave interval. Basically, the seventh interval often appears as a component of the dominant seventh chord, and this interval itself (that is, two-note interval) appears much less frequently than the octave interval. Logically, it makes sense that the seventh interval would be used less often than other intervals, given that it is a dissonant interval. Therefore, the participants have had less experience seeing the seventh interval on its own, without having any other notes around it. In addition, as mentioned in section Differences between the Presented Intervals and the Played Intervals, since there were two interval conditions in this experiment, it is also possible that there was a bias toward these interval errors.

We categorized the positional errors into four types based on the geometric features and the appearance probability. All our results consistently indicated that these two factors are essentials in efficient score reading. These factors give a clue to speeded score reading as well as a cause to misreading. As mentioned in section Introduction, the skilled pianists must not only read a huge number of notes, they must also process disparate bits of information simultaneously and continuously. Therefore, even within the readable range, the skilled pianists would be likely to reduce the mental processing load required for sight-reading scores by perceiving patterns rather than reading individual notes. Such reduction of the processing load may contribute the increase of information processing capacity in musical expression and performance.

## Conclusion

The aim of this study was to investigate the visual information that pianists rely on when reading music. We measured the accuracy of the musical score reading of 16 skilled pianists and investigated its relationship with the geometrical features.

Pianists could easily read a single note when it was located within three ledger lines. This range may be determined by the familiarity (or appearance probability) in actual musical scores. The readable range of the octave interval was extended compared to that of the single note. The pianists were also able to recognize the correct interval even if they misread the height of the target note, suggesting that two notes composing the octave interval are perceived as a two-tone geometric pattern. We also showed the spatial relationship between note heads and ledger lines is an important factor to reading notes, and this relationship sometimes causes misreading. All these results indicate that pianists rely on geometric patterns of the notes, not logically counting the number of ledger lines, when reading scores: Tight visuo-motor coupling formed through the long experience must be the key mechanism to the fast score reading.

Though we dealt only with a single note and an interval in the present study, we can easily extend the present experiment to other types of notations such as chord, arpeggio, and melody. The present experimental method would contribute to reveal various factors for efficient score reading.

## Author contributions

EA designed the experiment, ran the experiment, analyzed the experimental data, and wrote the manuscript. She made the original proposal of this research and received a grant from JSPS for this topic. YS gave essential ideas on the experimental procedure. He gave the interpretation of the experimental result and revised the manuscript.

### Conflict of interest statement

The authors declare that the research was conducted in the absence of any commercial or financial relationships that could be construed as a potential conflict of interest.
