# Supplementary figures and images for: Visual Information Pianists Use for Efficient Score Reading
Source: Front Psychol. 2018 Nov 22;9:2192. doi: 10.3389/fpsyg.2018.02192 (PMC6261976; doi:10.3389/fpsyg.2018.02192)

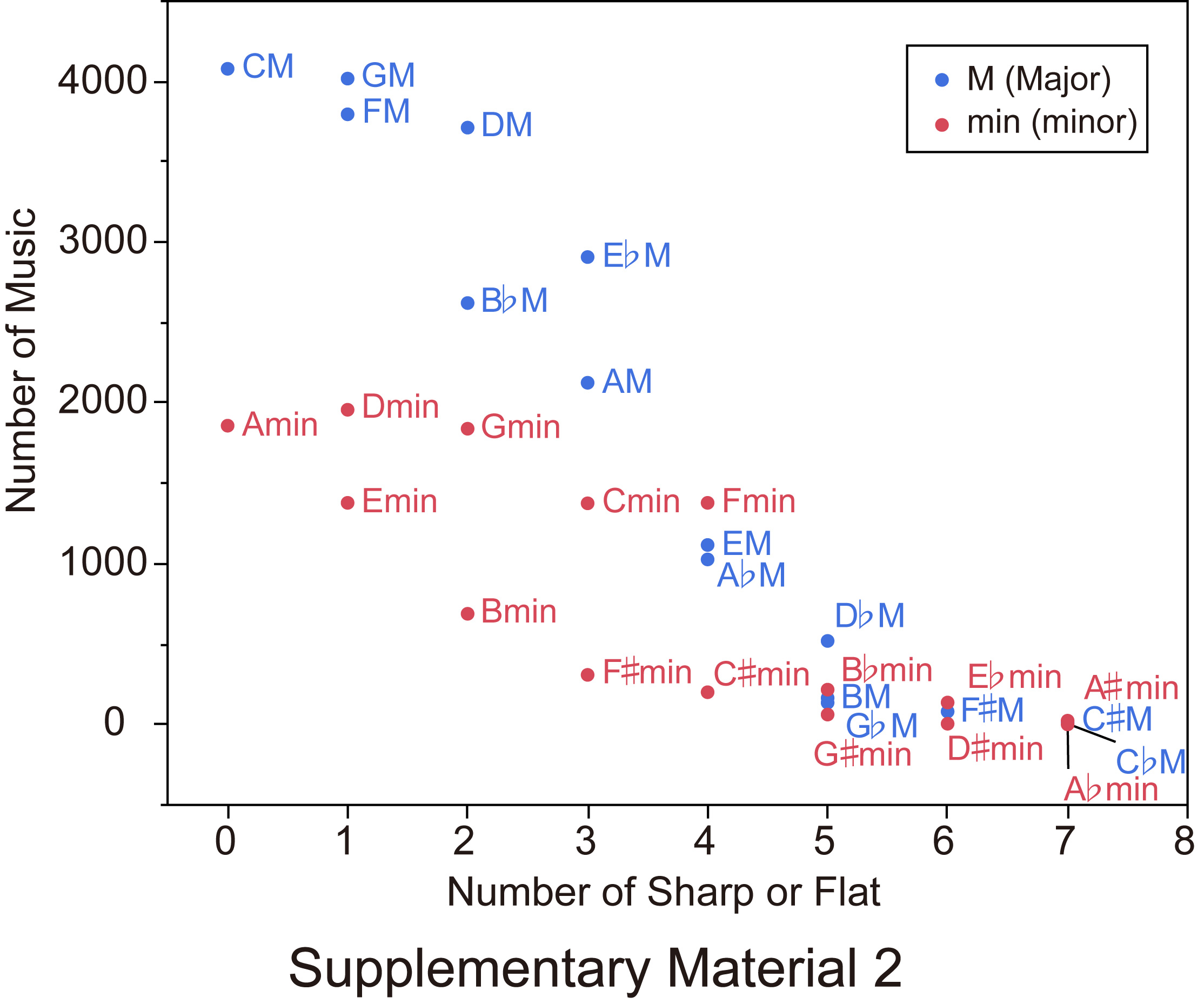

Supplement: Supplementary file 2 [file Image_1.jpg]
