# Supplementary material for: Visual Information Pianists Use for Efficient Score Reading
Source: Front Psychol. 2018 Nov 22;9:2192. doi: 10.3389/fpsyg.2018.02192 (PMC6261976; doi:10.3389/fpsyg.2018.02192)
Supplement: Supplementary file 3 [file Data_Sheet_1.DOCX]

# Supplementary material 3

We will review each target note in a practical way and especially discuss the details about the positional errors that are considered to be Type II, Type III, and Type IV, and that are exceptional.

#### Single Note

##### C Note

For C notes, the cause of positional errors was categorized as either Type I, Type II, or Type IV (Supplementary 3a).

[Type II] C♭ and C were often misread as either B♭ or D♭, and either B or D, respectively. As mentioned above, the impression for the ledger line configuration of familiar height of C notes is considered to be on the ledger line. However, in the range that the participants gave positional errors as either D or B notes, C notes were written as a note above or below a ledger line. There is, therefore, the possibility that the participants misread it as either B♭ or D♭, or B or D, which are located on the ledger line within the frequently used range. In the case of C♭, since it was necessary for the participants to play a white key even if it had an accidental mark, there is the possibility that it is Type III. However, it is not possible to explain the reason C without flat was incorrectly answered as either B or D.

[Type IV (Type II & Type III)] At first glance, it seems that the reason C♯ was incorrectly answered as A♯ is a Type I error. Actually, because six participants misread C7 on the G-clef as A6, some of them might have had the correct impression for the ledger line configuration. However, it cannot be the only reason since C♭, C, and C♯ have identical geometrical features, except for the existence of an accidental mark. As with the case of C♭ or C, there is the possibility of the participants having the impression of them being B♯ or D♯. As a matter of fact, in the case of C♯3 on G-clef, the participants misread it as D♯3. It is therefore assumed that although the participants initially judged the target note to be B♯ from the result of pattern matching with geometrical features, they modified their judgements based on the appearance probability, and finally misread C♯ as A♯.

##### D Note

For D notes, the cause of positional errors was categorized as either Type I or Type IV (Supplementary 3b).

[Type I] All D notes were often misread as either B or F notes. In both cases, the ledger line configuration is the same.

[Type IV (Type I & Type III)] Six participants misread D♭3 on G-clef as E♭3. When considering that the participants often misread D3 as F3, it can be assumed that they initially had an impression of D♭3 being F♭3. However, F♭3 is the note with accidental mark that indicates the need to play the white key. There is, therefore, the possibility that the participants modified their judgements based on the appearance probability, and finally misread D♭3 as E♭3.

Five participants misread D♯1 on F-clef as C♯1. It is also assumed that although the participants initially had an impression of D♯1 being B♯1, they modified their judgements based on the appearance probability, and finally misread D♯1 as C♯1.

##### E Note

In E notes, it seemed that the cause of positional errors differed depending on their height (Supplementary 3c).

[Type II**]** Six participants misread E1 on the F-clef as D1. The impression for the ledger line configuration of familiar height of E notes is considered to be on the ledger line. The ledger line configuration of D1 is on the ledger line, and it corresponds to the participants’ incorrect impression of the ledger line configuration of E1. This might have caused confusion for the participants.

[Type II (or III**)]** Four participants misread E♯1 on the F-clef as F♯1. At first glance, it seems that this incorrect response was due to a Type III error, because E♯1 is the note with an accidental mark indicating the need to play the white key. Actually, some of the participants might have had an incorrect response due to a Type III error. There is, however, the possibility that the participants were misled by the impression of the ledger line configuration of the familiar height of E notes, considering that the participants often misread E1 as D1. Although the number of participants who gave the incorrect response did not reach four, three participants misread E♯1 on the F-clef as D♯1. This result may support the inference that this was a Type II error.

[Type III**]** Five participants misread E♯3 on the G-clef as F♯3 and five participants misread E♭3 on the G-clef as G♭3. Although the geometrical features of E3 on the G-clef are identical with E♯3 and E♭3, except for the existence of an accidental mark, there were a few incorrect responses. This means that the participants did not have the incorrect impression for the ledger line at the height of the E note, and E♯3 is the note with an accidental mark the instructed them to play the white key. It is therefore assumed that the participants gave positional errors based on the estimation of the appearance probability.

[Exceptions**]** In the case of E7s on the G-clef, since this note was the highest among all the target notes, most of the participants were unable to read it. Therefore, the positional errors were distributed in various notes. Especially in the case of E♯7, although 15 participants gave an incorrect response, there was no case where the incorrect answers were note concentrated. In this height, there is much uncertainty concerning the cause of the positional errors.

However, 6 participants out of 14 who gave an incorrect response misread E♭7 as D♭7. When considering that 4 participants out of 12 who gave an incorrect response misread E7 as C7, it can be assumed that they initially had an impression of E♭7 being C♭7. C♭7 is the note with an accidental mark instructing them to play the white key. It is therefore assumed that the participants modified their judgements based on the appearance probability, and finally misread E♭7 as D♭7.

##### F Note

In F notes, the cause of positional errors was categorized as either Type I or Type IV (Supplementary 3d).

[Type I**]** F notes were mainly misread as the note on the same ledger line configuration.

[Type IV (Type I & Type III)**]** F♭ notes were mainly misread as A♭. F♭ is the note with an accidental mark instructing them to play a white key. It is therefore assumed that the participants modified their initial judgement based on its appearance probability and ledger line configuration. We should note that the notes of exactly the same height either without an accidental mark or with sharp (i.e. F♯4 and F♯3) were correctly read in almost all the cases.

##### G Note

Since all the G notes at any height are relatively close to the staff, there was only a small number of positional errors (Supplementary 3e).

[Type I**]** However, G♭6 and G6 were often misread as E♭6 and E6, respectively. These are the same ledger line configuration.

[Type III**]** Four participants misread G♭1 on the F-clef as A♭1. When A♭ and G♭ are compared, it seems that A♭ should appear more frequently based on the key signature. While A♭ appears in a key signature with three accidental marks, such as E♭ Major, G♭ appears in a key signature with five accidental marks, such as D♭ Major.

[Exceptions**]** Seven participants misread G♯1 on the F-clef as F♯1. This cannot be explained from the viewpoint of its appearance probability; both F♯ and G♯ appear very frequently. In addition, G1, which does not accompany any accidental mark, was correctly answered in most cases. The most significant clue is the fact that the geometrical features on both G♯1 on the F-clef E♯1 on the G-clef are exactly the same. E♯1 is the note with an accidental mark instructing them to play the white key. It is therefore assumed that the participants modified their initial judgements based on its appearance probability, and finally misread G♯1 as F♯1. However, this interpretation is just one of the possibilities, because there were few identical incorrect responses in other G1 notes.

##### A Note

Since all the A notes at any height are relatively close to the staff, there was only a small number of positional errors (Supplementary 3f).

[Type I**]** However, A1 and A♯4 were often misread as C1 and F♯4, respectively. These are the same ledger line configuration.

[Type III**]** A♯ was misread as G♯. When considering that both A and A♭ were correctly answered in most cases, it is difficult to conclude that the positional errors were caused by the geometrical features. When A♯ and G♯ are compared, it seems that G♯ should appear more frequently based on the key signature. More specifically, A♯ and G♯ appear in the key signature with five (B Major) and three (A Major) accidental marks, respectively (see Supplementary material 2). Although A♯often appear music in B Minor as an accidental mark, the number of musical works written in these key signatures is relatively low.

##### B Note

In B notes, the cause of positional errors was categorized as either Type I, Type II, or Type IV (Supplementary 3g).

[Type II**]** B and B♯ were often misread as either A or C and either A♯ or C♯, respectively. The impression for the ledger line configuration of the familiar height of B notes is considered to be above/below the ledger line. However, in the range that the participants gave positional errors as either A or C notes, B notes were written as a note on a ledger line. There is, therefore, the possibility that the participants misread it as either A or C, and either A♯ or C♯, which are located above/below the ledger line within the frequently used range. In the case of B♯, since it was necessary for the participants to play a white key even if it had an accidental mark, there is the possibility that it this was a Type III error. However, it is not possible to explain the reason that B without an accidental mark was incorrectly answered as either A or C. This result may support that this was a Type II error.

[Type IV**]** B♭0 was often misread as D♭0. As mentioned above, B0 and B♯0 on the F-clef have identical geometrical features, but were misread as A0 or C1, or A♯0 or C♯1. This cause of positional errors is Type II. It can be assumed that the participants initially had an impression of B♭0 being either A♭0 or C♭1. However, C♭1 is the note with an accidental mark instructing them to play the white key. There is, therefore, the possibility that the participants modified their judgements based on the appearance probability, and finally misread B♭0 as D♭0. In addition, as there is no key of A♭0, the incorrect responses were concentrated on D♭0.

### Octave Interval

[Type I**]** There were many cases where the participants misread the notes closer to the staff contained in the octave interval as the note of same ledger line configuration.

[Type III**]** B♯6 on the G-clef was misread as F♯6. Since B♯6+ B♯7 was the highest among all the target notes in the octave-interval condition, most of the participants were unable to read it. The incorrect responses were distributed among various notes. Actually, 4 participants out of 16 who gave an incorrect response misread B♯6 on the G-clef as F♯6. In this height, there is much uncertainty about the cause of the positional errors.

[Type IV**]** The 4 participants out of 16 who gave an incorrect response misread B♭6 on the G-clef as A♭6. As mentioned above, this was also the highest among all the target notes in the octave-interval condition, and most of the participants were unable to read it. Therefore, the positional errors were distributed among various notes. However, when considering that B♯6 was misread as G♯6, it can be assumed that the participants initially had an impression of B♭6 being G♭6. As mentioned in G note of the single-note condition, when A♭ and G♭ are compared, it seems that A♭ should appear more frequently if based key signatures. There is, therefore, the possibility that the participants modified their judgements based on the appearance probability, and finally misread B♭6 as A♭6.

Five participants misread A♭6 on the G-clef as G♭6. When considering that A♯6 was misread as F♯6, it can be assumed that the pianists initially had an impression of A♭6 being F♭6. However, F♭6 is the note with an accidental mark that instructed them to play the white key. There is, therefore, the possibility that the participants modified their judgements based on the appearance probability, and finally misread A♭6 as G♭6.

### Seventh Intervals

[Type I**]** In case of the seventh interval, the target notes that are close to the staff were correctly played in most cases. Also, in the case of incorrect answers, the participants misread the target notes as the notes with the same ledger line configuration and the same geometrical characteristics.

[Exceptions**]** G1 and G♯1 on the F-clef were misread as A1 and A♯1 on F-clef. The G1+A0 and G♯1+ A♯0 were the lowest among all the target notes in the seventh-interval condition, and most of the participants were unable to read it. Therefore, the positional errors were distributed among various notes. In addition, A0 is the lowest key on the piano.


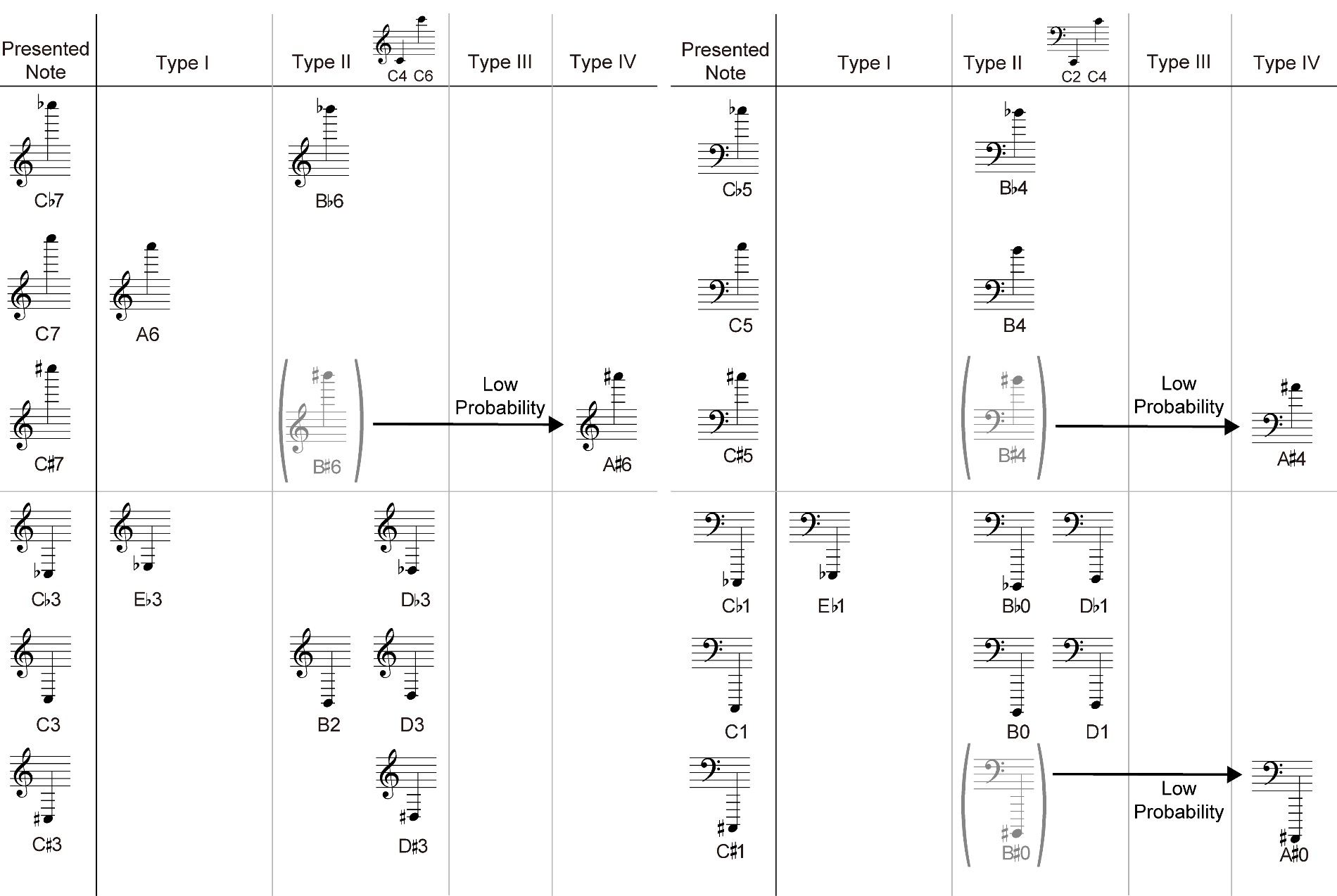


# Supplementary material 3a

Positional errors to the C note.


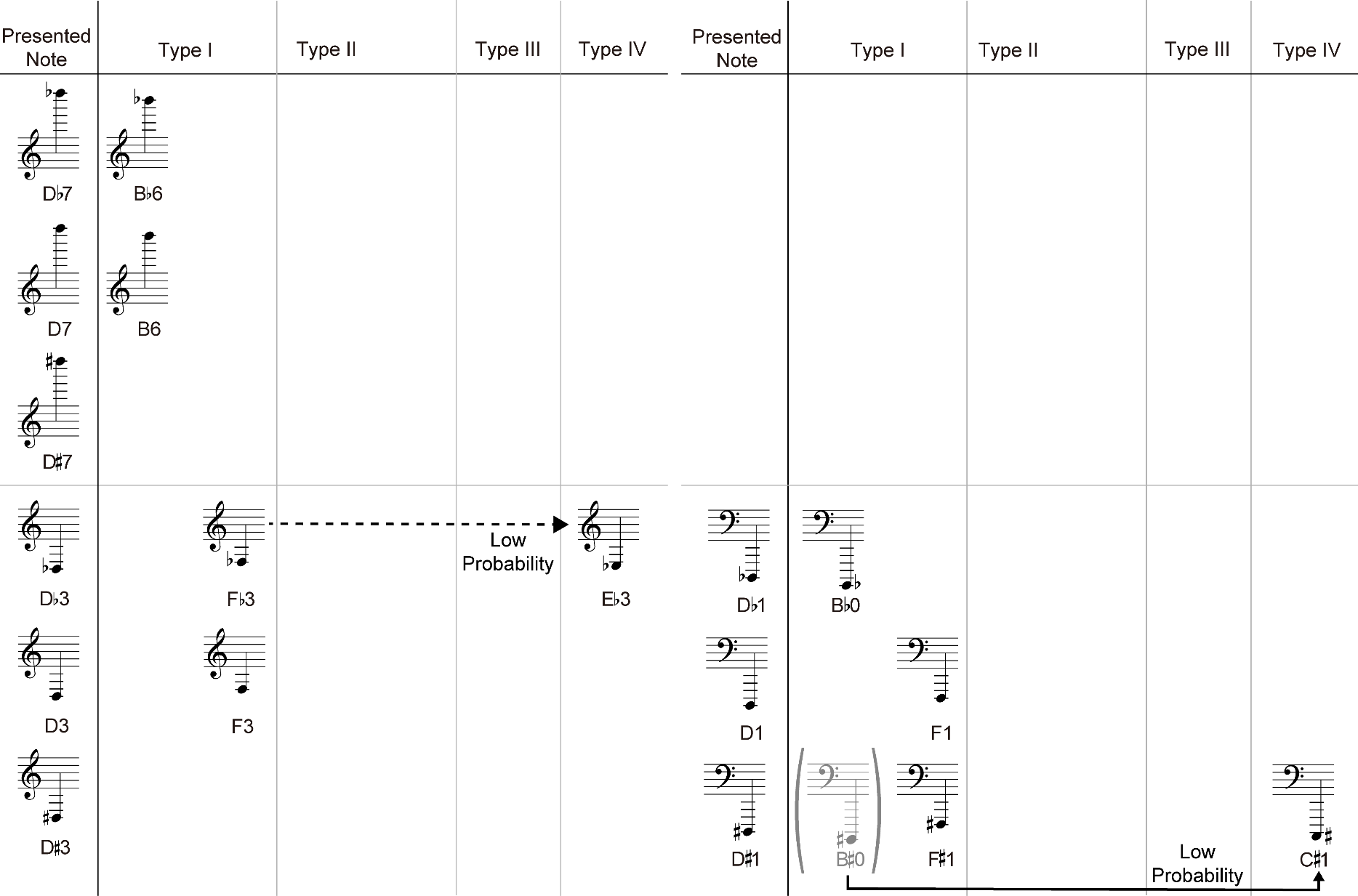


# Supplementary material 3b

Positional errors to the D note.


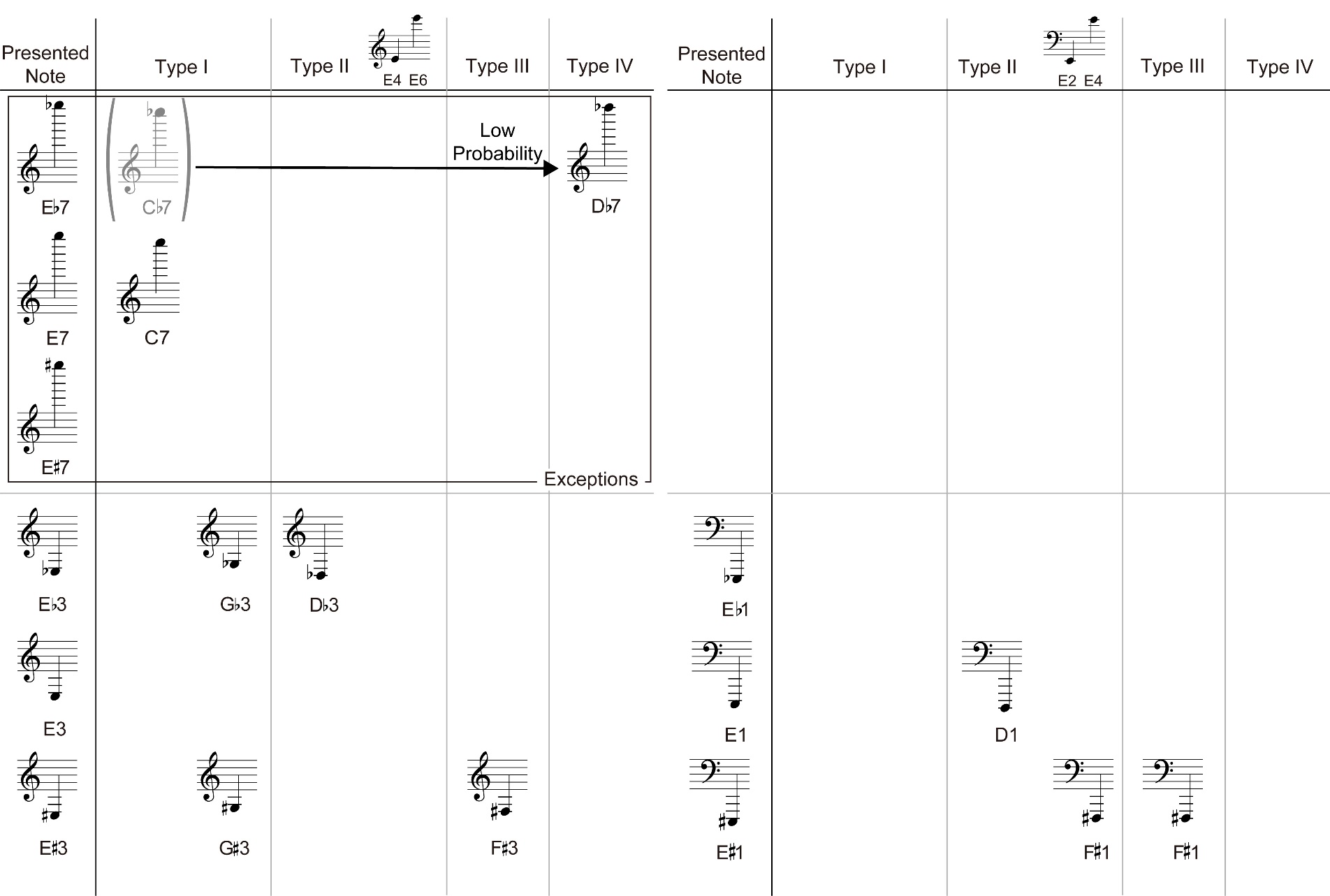


# Supplementary material 3c

Positional errors to the E note.


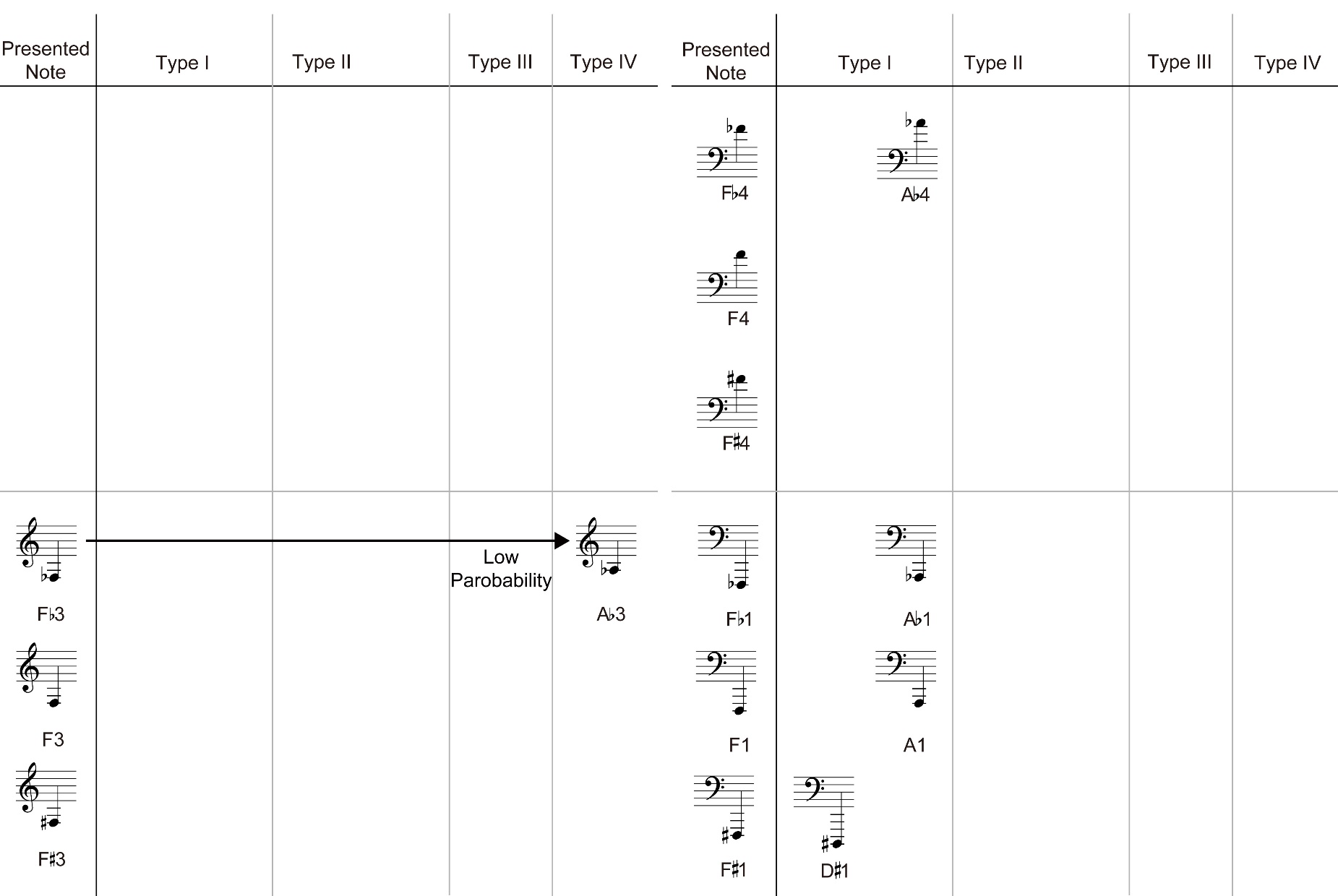


# Supplementary material 3d

Positional errors to the F note.


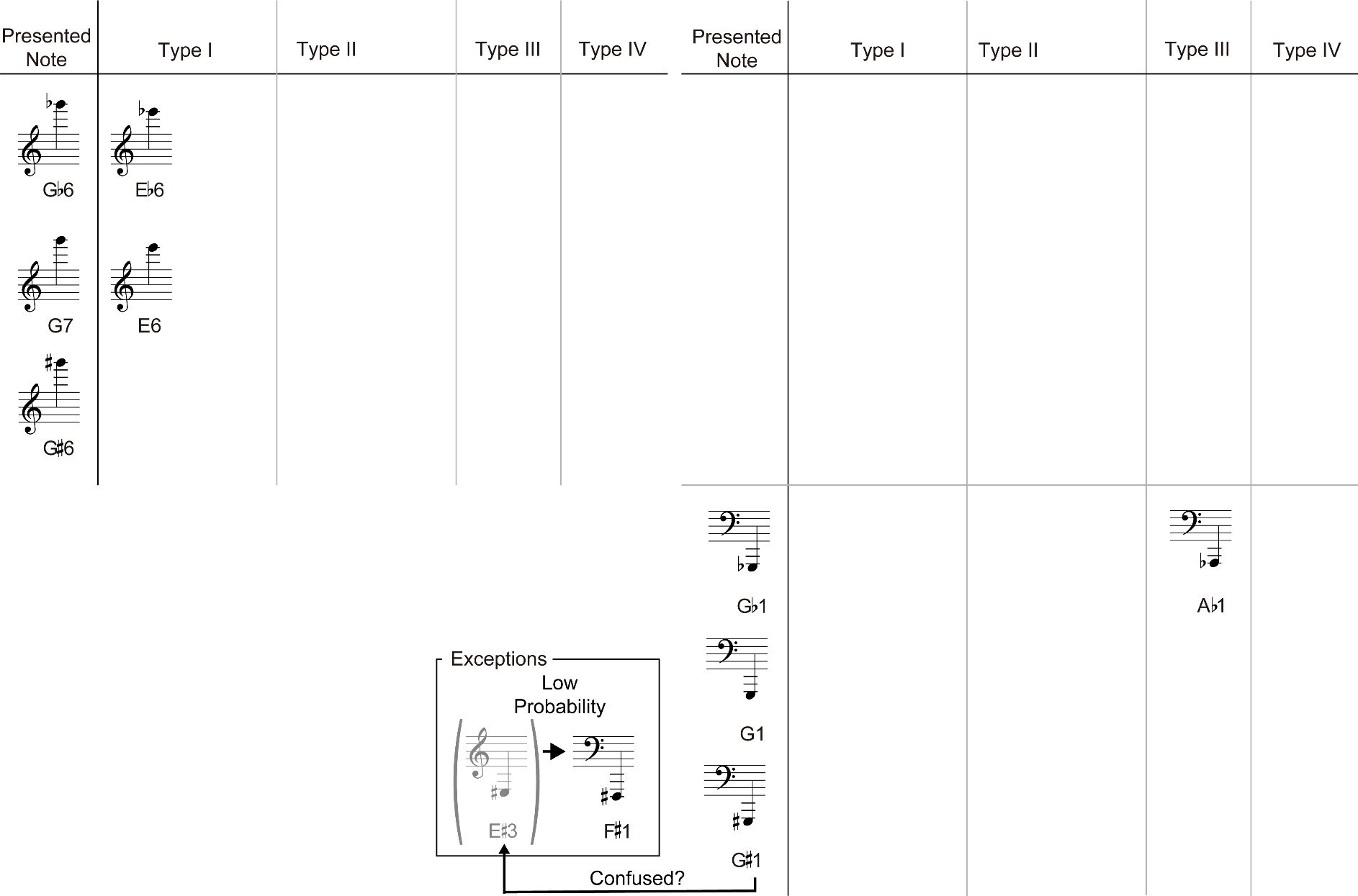


# Supplementary material 3e

Positional errors to the G note.


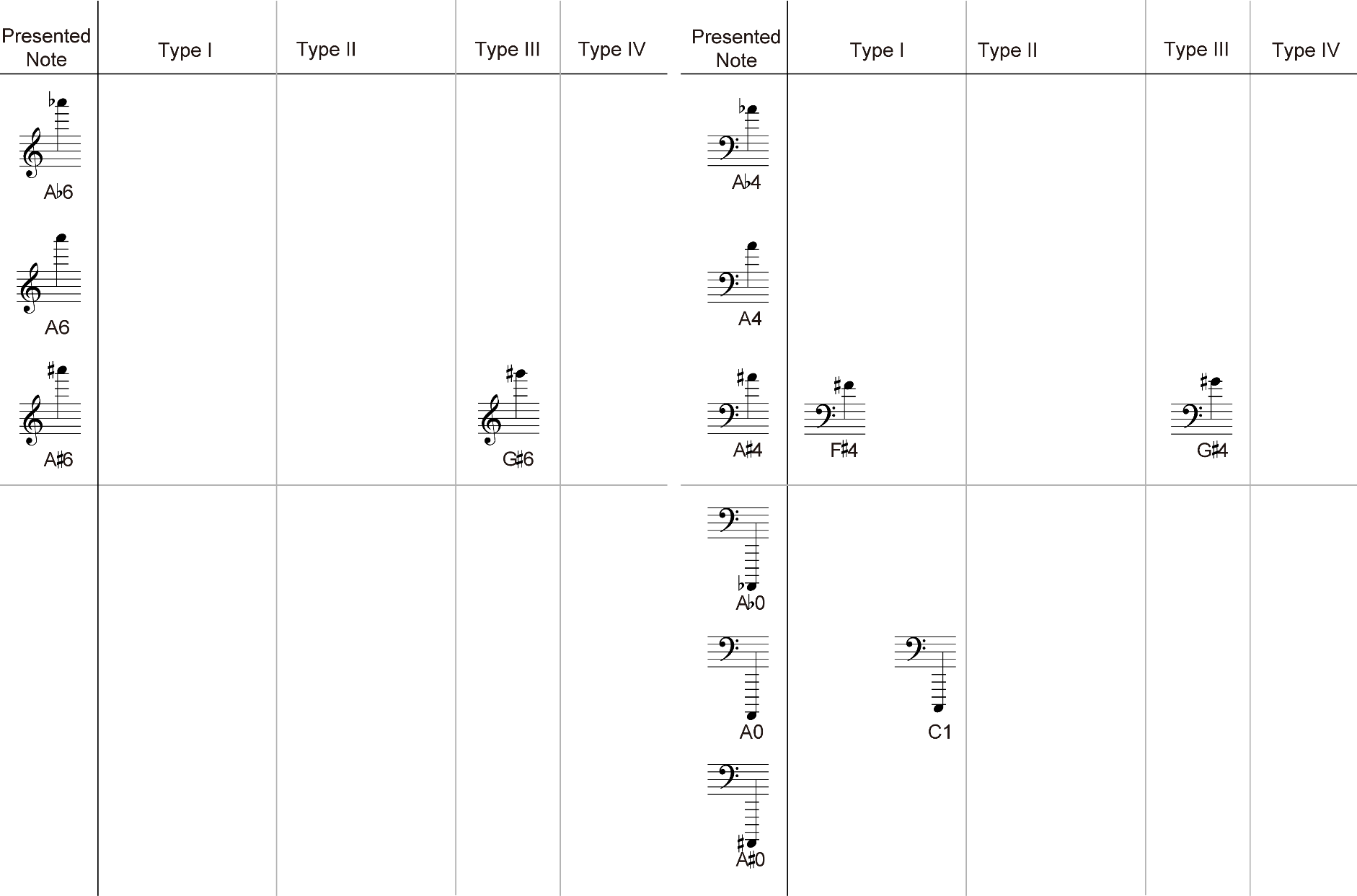


# Supplementary material 3f

Positional errors to the A note.


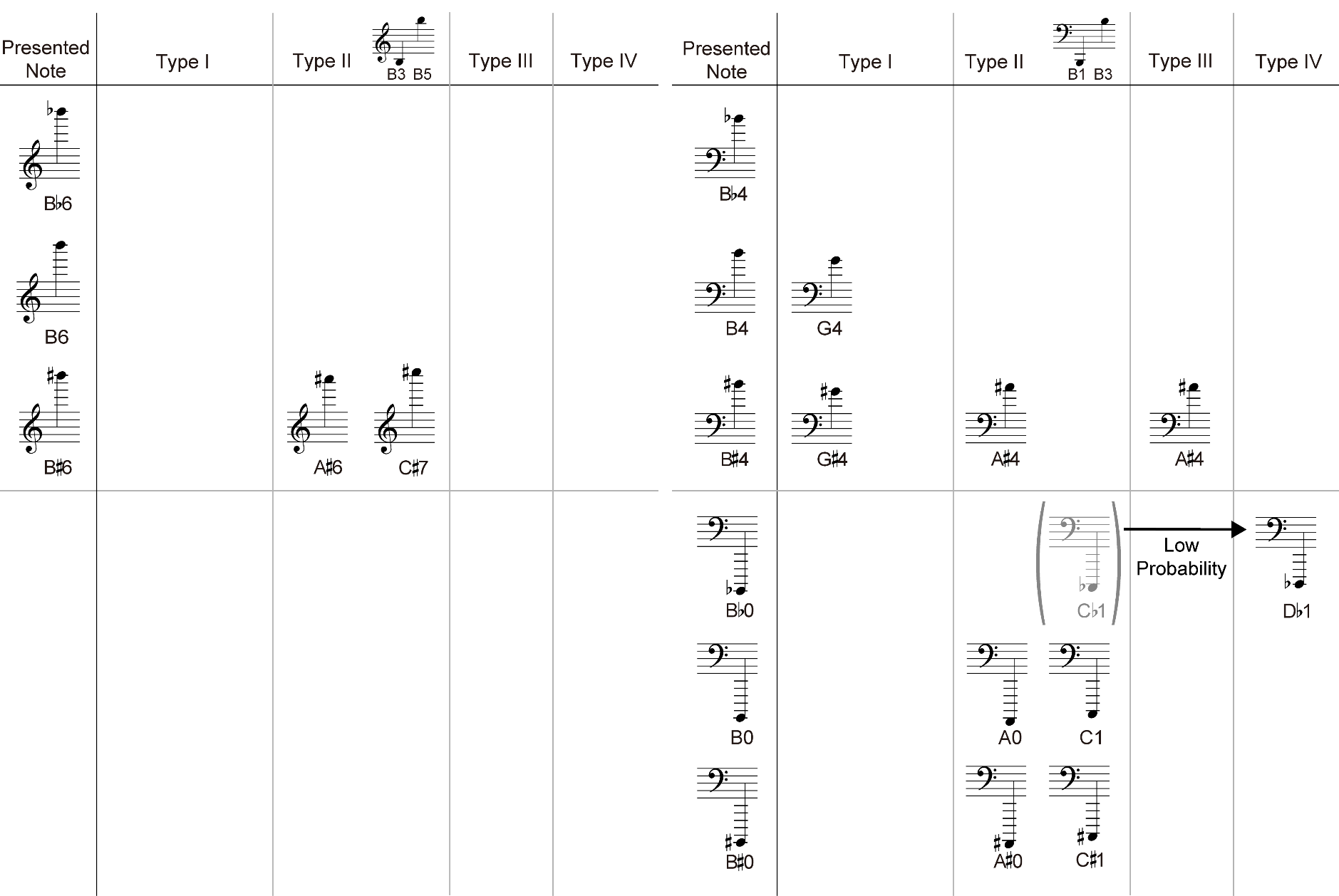


# Supplementary material 3g

Positional errors to the B note.
